# Cognitive Dysfunction and Dementia in Primary Sjögren's Syndrome

**DOI:** 10.1155/2013/501327

**Published:** 2013-09-19

**Authors:** Frederic Blanc, Nadine Longato, Barbara Jung, Catherine Kleitz, Laure Di Bitonto, Benjamin Cretin, Nicolas Collongues, Christelle Sordet, Marie Fleury, Vincent Poindron, Jacques-Eric Gottenberg, Olivier Anne, Dan Lipsker, Thierry Martin, Jean Sibilia, Jérôme de Seze

**Affiliations:** ^1^Neuropsychology Unit, Service of Neurology, University Hospital of Strasbourg, 1 avenue Molière, 67000 Strasbourg, France; ^2^ICube Laboratory and Strasbourg Federation of Translational Medicine (FMTS), University of Strasbourg and CNRS, Strasbourg, France; ^3^Memory Resource and Research Centre (CMRR) from Alsace, University Hospital of Strasbourg, 1 avenue Molière, 67000 Strasbourg, France; ^4^Department of Rheumatology, University Hospital of Strasbourg, 1 avenue Molière, 67000 Strasbourg, France; ^5^Department of Internal Medicine and Immunology, University Hospital of Strasbourg, 1 avenue Molière, 67000 Strasbourg, France; ^6^Department of Neurology, Hospital of La Rochelle, La Rochelle, France; ^7^Department of Dermatology, University Hospital of Strasbourg, Faculty of Medicine, University of Strasbourg, Strasbourg, France; ^8^National Referral Center for Autoimmune Diseases, University Hospital of Strasbourg, 1 avenue Molière, 67000 Strasbourg, France

## Abstract

*Background*. Primary Sjögren's syndrome (PSS) is a frequent systemic autoimmune disease. In this study, we aimed to explore the cognitive impairment and the correlations with brain MRI. *Methods*. Twenty-five patients (mean age 55 ± 11.8 years, 21 females) with PSS were prospectively selected and tested with a French translation of the Brief Repeatable Battery for Neuropsychological Examination. The results were compared with the scores for 25 matched patients with multiple sclerosis (MS) and 25 controls. Brain lesions were assessed by brain MRI using the Wahlund classification. *Results*. Fifteen of the 25 PSS patients (60%) presented with cognitive disorders versus 19/25 MS patients (76%). Five patients had dementia in the PSS group. Speed of information processing, attention, immediate and long-term memory, and executive functions were frequently impaired. The mean duration of cognitive complaints was 5.6 ± 6.1 years, and the mean duration of PSS was 15.8 ± 14.0 years. A trend towards a correlation was found between the severity of cognitive impairment and the degree of white matter lesions (WML) (*P* = 0.03, rho = 0.43). *Conclusion*. Cognitive impairment—mild or dementia—exists in patients with PSS. Further MRI studies are needed to better understand the precise neural basis of cognitive impairment in PSS patients.

## 1. Background

Primary Sjögren's syndrome (PSS) is a frequent systemic autoimmune disease, characterized by mononuclear infiltration of salivary and lachrymal glands, leading to xerostomia and xerophthalmia (sicca syndrome). Neurological manifestations are observed in 8.5−42% of cases [1]. Delalande et al. reported central nervous system involvement in 56 out of 82 patients (68%) with PSS and neurological manifestations [2]. Cognitive manifestations have been observed in 10–50% of PSS patients [3–8]. Few studies have described the cognitive pattern of PSS [4, 5].

This study examined PSS patients and compared the cognitive pattern and the severity of cognitive impairment to those seen in patients with multiple sclerosis (MS) and the correlations with brain lesions on magnetic resonance imaging (MRI).

## 2. Patients and Methods

Twenty-five consecutive patients (mean age 55 years ± 11.8 years, range 30–74, 21 women and 4 men) with PSS diagnosed according to American-European Consensus Group criteria [9] were evaluated prospectively at the Neuropsychology unit of the University Hospital of Strasbourg from January 2008 to April 2012. Six patients were diagnosed for PSS at the Neuropsychology unit the others were diagnosed for PSS previously, because of rheumatological and/or neurological symptoms. Thus, among these 25 patients, neuropsychological symptoms came first for 6 patients, neurologic symptoms for 11, and rheumatologic for 8.

Patients were tested with the BCcogSEP, a French translation of the Brief Repeatable Battery for Neuropsychological Examination, including 14 subtests [10]. The results of PSS patients were compared with the standard for each test and with the scores for 25 MS patients diagnosed according to the McDonald criteria [11] and with 25 controls matched for age, sex, and educational level. Controls were recruited using advertising in a local newspaper.

MS patients have been chosen to be compared to PSS because: (1) MS patients have cognitive impairment in about 50% to 70% of cases: the majority of MS patients have a mild cognitive impairment [12]; (2) MS patients can have also dementia as described previously [13]; (3) PSS is an autoimmune disease, and we wanted to compare this autoimmune disease to another one; MS is a common autoimmune disease of the central nervous system; (4) it was less scientifically accurate to compare PSS to Alzheimer's disease (AD) because of the difference of median age between PSS (about 50–60) and AD (about 80).

Pain due to arthralgia and/or neuropathy might have affected performances in PSS patients. This is the reason why PSS patients were not tested during relapses, to avoid such.

All MS and PSS patients have had complete clinical examination and an expanded disability status scale (EDSS) evaluation [14].

Dementia was diagnosed according to *Diagnostic and Statistical Manual of Mental Disorders, Fourth Edition, Text Revision (DSM-IV-TR)*. Mild Cognitive Impairment (MCI) was diagnosed according to Petersen [15], and MCI was classified as amnestic MCI and nonamnestic MCI, with single or multiple domain impairment [15]. Other causes for MCI and dementia were ruled out in the PSS group; thus, we verified that the patients did not fill the criteria of AD [16], Lewy body dementia [17], and frontotemporal dementia [18].

### 2.1. Neuropsychological Assessment

We assessed PSS, MS patients, and controls using* Batterie Courte d'évaluation cognitive destinée aux patients souffrant de Sclérose En Plaques* (BCcogSEP) [10]. The BCcogSEP is a battery of tests to evaluate cognitive functions and was specially designed for MS patients. It is the French modified version of the Brief Repeatable Battery of Neuropsychological tests for Multiple Sclerosis (BRB-N) proposed by Rao and comprises the 5 modified BRB-N tests: a selective reminding test (BCcog-SRT), a visuospatial memory test (10/36), the paced auditory serial addition test (PASAT), a verbal fluency test, and the digit symbol substitution test of the WAIS-R (DSST) [19]. The BCcog-SRT presents a list of 15 unrelated words. Upon administration of the first trial, participants are asked to recall as many words as possible. Over each of ten more trials, only the words missed on the previous trial are presented, but participants are asked to recall as many words as possible. Following a 20-minute delay,  patients are again asked to recall as many words from the list as possible. If the patient does not recall the 15 words at this delayed recall, a recognition task for each 15 words is given: patients are asked to indicate which word was to store among four words. Common indices are the mean number of words recalled during the first phase (called “mean number of words”), the percentage of words systematically recalled on each trial (called “learning”), and the number of words recalled after the delay interval or delayed recall (called “SRT-DR”). The 10/36 test presents a checkerboard of 36 compartments, with 10 pieces in specified locations. Participants are presented with this stimulus for ten seconds during each of the three consecutive trials. For each trial, a blank checkerboard is given to the participant with instructions to replicate the position of the 10 pieces (immediate recall). After a 10–15 min delay, participants are again asked to replicate the position of the 10 pieces (delayed recall).

The PASAT test presents orally a figure (number) every 3 seconds (PASAT 3 s) and every 2 seconds (PASAT 2 s) sixty times. The subjects are asked to add the figure they just heard with the figure they heard before.

The verbal fluency tests ask subjects to say as many words as possible from a category in 1 minute. The semantic fluency asks subjects to say the maximum of name of animals; in the phonemic fluency, the maximum number of words begins with letter p (except proper nouns).

The DSST test asks subjects to correctly code a series of figures (from 1 to 9) with the corresponding letter. The test is scored as the number of correct responses generated in 90 seconds.

Three tasks were added in order to provide additional information about working memory and executive functions: the crossed tapping test, a “Go-No-Go” test, and the WAIS-R digit span subtest.

For the crossed tapping test, subjects are given a stick and asked to listen to a sound recording. They are instructed to tap twice on the table with the stick when they hear a single, brief sound and to tap once when they hear two, consecutive brief sounds. Both the kinds of sound are mixed. Practice trial is run before starting the task, which comprises 40 trials.

 For the “Go-No-Go” test, subjects are asked to put the right hand on the desk and to listen to a sound recording. They are instructed to raise the hand when they hear a single brief sound and to let the hand on the desk when they hear two consecutive brief sounds. Both the kinds of sound are mixed. Practice trial is run before starting the task, which comprises 40 trials.

Cognitive impairment was considered if four or more of the 14 subtests were inferior to the 5th percentile, as described by Dujardin et al. [10].

The Beck Depression Inventory (BDI) was added to detect depressed patients (score >10) [20].

### 2.2. Brain MRI Assessment

Brain MRI (T1, T2, and T2-FLAIR-weighted sequences) was performed in all patients. White matter lesions load was scored from axial FLAIR images using the semiquantitative method of Wahlund et al. (0 : no lesion, 1 : focal lesions, 2 : beginning confluence of lesions, 3 : diffuse involvement of the entire regions (with or without involvement of U fibers) [21].

### 2.3. Statistical Analysis

Statistical analysis was carried out using the Kruskal-Wallis nonparametric test and Mann-Whitney *U* nonparametric test to compare the numerical values for each cognitive test between the three groups. We looked for correlation between cognitive dysfunction and the following parameters: disease duration (beginning of the sicca symptoms), cognitive complaint duration, treatments, and fin spaces white matter lesions load according to Wahlund et al. [21]. For this statistical analysis, we used the Spearman rank correlation test. To correct for multiple comparisons, a *P* value ≤ 0.01 was considered statistically significant, a *P* value between 0.01 and 0.05 was considered as a trend, and a *P* value > 0.05 was considered not significant. Data were examined using SPSS 18.0.

## 3. Results

All patients with PSS had sicca symptoms. Twenty patients had abnormal histopathology of the minor salivary gland, and eight had Ro/SSA antibodies (Table 1). Eight patients had suffered from arthralgia. Eleven patients had previous other neurological symptoms: neuropathy (*n* = 2), optic neuritis and myelitis (*n* = 1), myelitis (*n* = 4), brainstem inflammation (*n* = 1), optic neuritis (*n* = 2), and seizures (*n* = 1)—this patient with encephalopathy and partial complex seizures with a favorable outcome under IV-IgG and immunosuppressive treatment was previously described in the literature and tested here two years after improvement [22]. The majority of the patients (81%) have had a CSF study, and among them 3 patients had oligoclonal IgG bands. Seven PSS patients (3 with benzodiazepine, one with benzodiazepine and antidepressant, one with antidepressant, and two with antiepileptics), 8 MS patients (4 with benzodiazepine, one with benzodiazepine and antidepressant, 2 with antidepressant, and one with antiepileptics), and 6 healthy subjects (4 with benzodiazepine, one with benzodiazepine, and antidepressant, and 1 with antidepressant) had psychoactive drugs.

Fifteen of the 25 PSS patients (60%) had cognitive impairment versus 19/25 MS patients (76%). Twenty-four PSS patients (96%) had at least one subtest below the 5th percentile. One patient among the 10 patients without cognitive disorders (i.e., number of subtests inferior to 5th percentile <4) had a BDI superior to 10 with a score of 28. All MS patients had at least one subtest below the 5th percentile. There was no significant difference between the PSS and MS groups for any of the 14 subtests of the BCcogSEP, and there was a significant difference between PSS and controls and between MS and controls for all subtests of the BCcogSEP, except for backward digit span and Go-No-Go (Table 2). Recognition task of the SRT (not shown on Table 2) was done correctly by patients and controls.

 Among the 15 PSS patients with cognitive disorders, ten had an MCI and five a dementia. Among the PSS patients with MCI, five had a nonamnestic single domain and five an amnestic MCI multidomain. Among the 19 MS patients with cognitive disorders, fifteen had an MCI. Among the MS patients with MCI, twelve had an amnestic MCI multidomain, two a non-amnestic single domain, and one an amnestic MCI single domain. Five patients with PSS (two women and three men, mean age of 62 ± 10 years) and four patients with MS (two women and two men, mean age of 55 ± 13 years) had dementia. All of these demented patients had more than 7 subtests below the 5th percentile. Four demented PSS patients had also myelitis and three of them IgG oligoclonal bands.

An analysis of cognitive functions of the PSS patients showed a predominance of cognitive disorders in speed of information processing (DSST, PASAT), verbal memory (learning and immediate recall of SRT), attention (forward digit span, immediate recall of SRT), and executive functions (PASAT, fluencies) (Table 2). Six patients had isolated (i.e., without other neurological symptoms) definite cognitive dysfunctions (i.e., number of subtests inferior to 5th percentile ≥4) including 4 patients with arthralgia. Nine patients among the 25 were recognized to have impaired cognitive functions, when they were initially diagnosed with PSS.

The mean duration of cognitive complaints was 5.6 ± 6.1 years (from 1 to 26 years), and the mean duration of Sjögren's syndrome was 15.8 ± 14.0 years (from 1 to 34 years).

**Figure 1:**
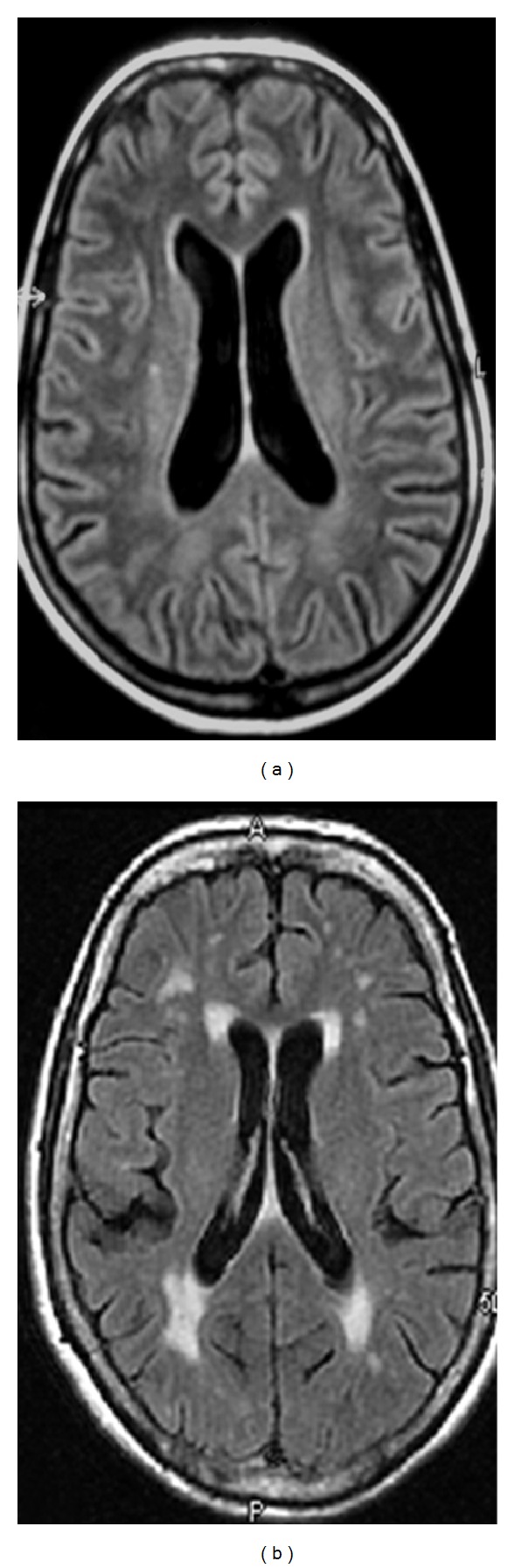
Axial brain FLAIR-magnetic resonance imaging of two female patients with primary Sjögren's syndrome. Patient (a) had three cognitive subtests below the 5th percentile and a Wahlund score of 1 for white matter lesions. Patient (b) had five cognitive subtests below the 5th percentile and a Wahlund score of 2 (beginning confluence of lesions) for white matter lesions.

**Figure 2:**
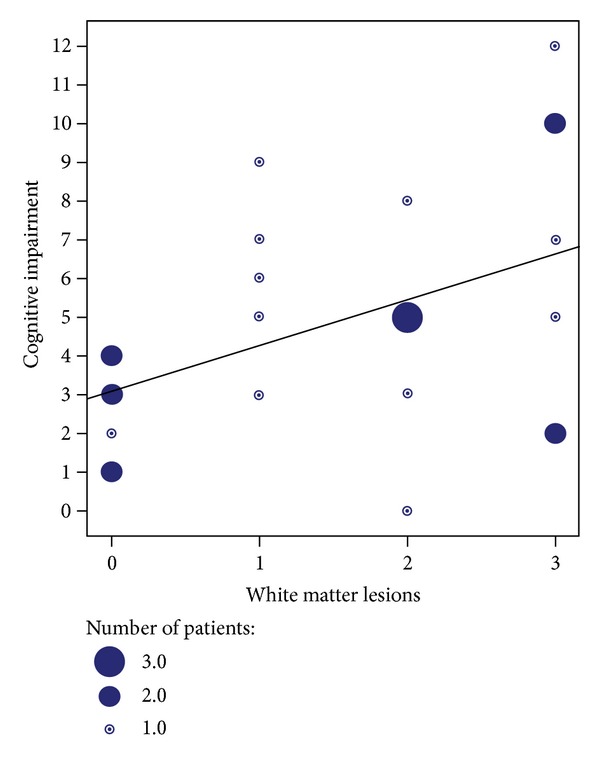
Scatter plot. Cognitive impairment (number of subtests inferior to the 5th percentile among 14) against white matter lesions load (Wahlund classification from 0 to 3). Spearman rank correlation coefficient was rho = 0.43 (*P* = 0.03); linear curve adjustment (*R* ^2^ = 0,205).

A trend towards a correlation was found between the intensity of brain white matter lesions and the severity of cognitive dysfunctions (*P* = 0.03, rho = 0.43) (Figures 1 and 2). All PSS demented patients had a Wahlund score of 2 or 3. However, six patients out of ten with MCI had only a few brain lesions (Wahlund score 0 to 1).

For PSS and MS patients, no correlation was found between the severity of cognitive dysfunctions and age, duration of cognitive complaints, duration of the disease, treatments, and EDSS.

## 4. Discussion

This study shows that, among PSS patients, 60% have cognitive impairment, including patients with dementia. Moreover, a trend towards a correlation has been found between cognitive impairment and WML.

Cognitive functions were tested in six previous studies [3–8]: in three studies, all patients have been tested [3, 5, 8], and four studies gave details of the cognitive results [3–5, 8]. In the study of Malinow et al. [4], 16 out of 40 patients were tested, and seven were found to have mild cognitive impairment, mainly of attention functions. Lafitte et al. [5] tested 10 out of 36 patients with neurological PSS and found cognitive impairment with executive dysfunction and attention deficit in eight. Mataró et al. tested 15 patients and found cognitive impairment related to memory and frontal lobe functioning in seven [3]. Finally, Segal et al. tested 39 PSS patients, including 20 PSS patients with cognitive complaints, and found psychomotor speed processing and verbal reasoning impairment [8]. Here, we found PSS patients with cognitive slowing (DSST, PASAT), long-term memory impairment (SRT, 10/36 spatial recall test) but with a good recognition, immediate memory impairment (forward digit span), attention dysfunction (PASAT, digit span), and executive impairment (PASAT, fluencies). This subcortical cognitive impairment seen in PSS patients seems to be similar to that observed in MS patients but is less frequent.

We found also a trend towards a correlation between global cognitive dysfunction and brain WML. Previously, psychomotor speed processing was correlated with WML and attention with ventricular volume, in PSS patients [3]. However, it appears that WML in PSS patients do not differ from those in healthy subjects [23]. Thus, in our cohort, 50% of our PSS patients without significant brain lesions (Wahlund score 0 to 1) had cognitive disorders. The mechanism of cognitive impairment for such patients is quite unclear. In the same way, radiological presentation of articular manifestations (AMs) in PSS is rarely erosive; thus, among 188 patients with PSS and AMs, 185 patients had normal radiography of the articulations [24]. The possibility of a too discrete inflammation in brain of patients with PSS and cognitive impairment to be detected with standard brain MRI is to be considered. Recently, Segal et al. have demonstrated using diffusion tensor imaging (DTI) sequences in MRI that subtle frontal region white matter microstructures alterations accompanied cognitive impairment in PSS [25].

 Using functional imagery, Le Guern et al. have shown a correlation between decreased brain perfusion on ^99m^Tc-ECD brain SPECT and cognitive impairment [7]. More neuroimagery studies are now needed to better understand the neural basis of cognitive dysfunctions in PSS.

PSS dementia was previously but rarely described [26]. Here, we found 5 patients (33% of PSS patients with cognitive impairment) with dementia, and most of them had myelitis. Subcortical dementia in a middle-aged patient is usually due to vascular brain involvement and synucleinopathy diseases (i.e., Parkinson's disease and Lewy body disease) [27], and we showed here that it can be due to PSS.

The mean duration between the onset of sicca syndrome and cognitive complaints is about 10 years in our study. Excluding sicca syndrome, symptoms of PSS—as arthralgia—appear insidiously [28]. The cognitive complaint before cognitive testing is more than 5 years in our study: partly probably because of the insidious apparition. Thus, it is of high importance that clinicians look for sicca syndrome when speed processing, attention, and memory and/or executive dysfunctions are detected on neuropsychological tests, even if brain MRI is normal or with few WML. However, no treatment guidelines do exist concerning cognitive disorders in PSS.

Because PSS is a frequent autoimmune disease, general practitioners and specialists have to be informed of the possibility of cognitive impairment due to PSS, including dementia.

Subcortical cognitive impairment in PSS patients and a trend towards a correlation with brain MRI WML need to be confirmed in larger studies. Longitudinal prospective studies in PSS patients with cognitive impairment using multimodal MRI are required to better understand the outcome of cognitive deficits, the origin of such deficits, and the potential benefit of treatment with immunomodulatory or immunosuppressive drugs.

## Figures and Tables

**Table 1:** Characteristics of patients with primary Sjögren's syndrome, multiple sclerosis, and controls.

Characteristics	Patients with PPS (*n* = 25)	Patients with MS (*n* = 25)	Controls (*n* = 25)	*P* value
Sex, F/M, number	21/4	21/4	21/4	>.05
Age, years	55 (11.8)	54 (10.3)	54 (9.4)	>.05
Educational level	12 (4.2)	12 (2.2)	12 (3.3)	>.05
Disease duration	15.5 (13.8)	18.0 (9.0)	NA	>.05
EDSS	1.1 (2.1)	3.6 (1.6)	NA	**<0.0001**
Patients with the presence of anti-SS-A antibodies	8	NA	NA	NA
Patients with a positive Schirmer test	19	NA	NA	NA
Patients with abnormal biopsy of minor salivary gland	20	NA	NA	NA
Grade III	9	NA	NA	NA
Grade IV	11	NA	NA	NA
Patients with ocular and oral dryness	24	NA	NA	NA
Patients with immunomodulatory treatment	11	7	NA	>.05
Patients with immunosuppressive treatment	14	15	NA	>.05
Patient without any treatment	5	3	NA	>.05
Subjects with psychoactive drugs	7	8	6	>.05

EDSS: expanded disability status scale; Disease duration: for PSS, the beginning of the disease was considered as the beginning of the sicca syndrome; for MS patients, the beginning of the disease was considered as the first relapse; PSS: primary Sjögren's syndrome; MS: multiple sclerosis; NA: not applicable, NT: not tested. **Chisholm** and **Mason** classification (grading from I to IV) was used for the histological analysis of minor salivary glands. Numbers shown are means (standard deviation).

**Table 2:** Results of cognitive tests in patients with primary Sjögren's syndrome, multiple sclerosis, and controls.

Cognitive tests	Mean score (SD)			*P* value		
	Patient with PSS (*n* = 25)	Patient with MS (*n* = 25)	Controls (*n* = 25)	PSS versus controls	MS versus controls	PSS versus MS
Selective reminding test (SRT)						
Mean number of words	9.9 (2.4)	9.4 (2.2)	11.6 (1.4)	.001	<.0001	NS
Learning	56.7 (24.5)	49.9 (24.1)	69.7 (16.4)	.047	.002	NS
SRT-DR (delayed recall)	12.0 (3.2)	11.6 (2.8)	13.7 (1.5)	.045	.003	NS
10/36 Spatial recall test						
Immediate recall	14.5 (6.2)	13.8 (4.7)	17.8 (4.7)	.049	.007	NS
Delayed recall	4.7 (2.3)	5.3 (2.2)	6.8 (2.4)	.001	.031	NS
Digit Span						
Forward	5.6 (1.9)	6.3 (2.0)	7.5 (1.3)	<.0001	0.042	NS
Backward	5.2 (1.7)	5.8 (1.9)	6.4 (2.1)	.03	NS	NS
Digit symbol substitution test (DSST)	44.8 (11.3)	43.1 (13.2)	61.5 (15)	<.0001	<.0001	NS
Paced auditory serial addition test (PASAT)						
3 s	34.7 (15.8)	32.7 (15.8)	46.7 (8.5)	.005	<.0001	NS
2 s	22.4 (13.6)	22.8 (13.8)	35.8 (9.6)	.001	.001	NS
Crossed tapping	3.9 (8.0)	3.1 (7.0)	0.4 (0.8)	.008	.031	NS
Go-No-Go	1.8 (3.0)	0.9 (1.1)	0.4 (0.6)	.047	NS	NS
Fluencies						
Phonemic	13.7 (5.0)	12.6 (5.5)	16.9 (5.4)	.036	.005	NS
Semantic	19.3 (4.7)	17.8 (5.4)	23.1 (4.4)	.011	<.0001	NS
**Nb subjects with 4 subtests <5e centile (%)**	15 (60)	19 (76)	0 (0)	<.0001	<.0001	NS

PSS: primary Sjögren's syndrome; MS: multiple sclerosis; Nb: number; NS: nonsignificant; SD: standard deviation.
